# Association of MMP-2 and hematological parameters with breast cancer metastasis: a cross-sectional study in Central Java, Indonesia

**DOI:** 10.3389/or.2026.1818926

**Published:** 2026-06-09

**Authors:** Alyssa Imani, Yan Wisnu Prajoko, Rudi Nirwantono, Fitya Syarifa Mozar, Bens Pardamean, Edward Kurnia Setiawan Limijadi

**Affiliations:** 1 Bioinformatics and Data Science Research Center, Bina Nusantara University, Jakarta, Indonesia; 2 Department of Surgical Oncology, Faculty of Medicine, Universitas Diponegoro, Semarang, Indonesia; 3 Department of Biotechnology, Faculty of Engineering, Bina Nusantara University, Jakarta, Indonesia; 4 Department of Clinical Pathology, Faculty of Medicine, Universitas Diponegoro, Semarang, Indonesia

**Keywords:** breast cancer, cross-sectional study, hematological parameters, metabolic parameters, metastasis, MMP-2

## Abstract

**Background:**

Breast cancer metastasis remains a major contributor to morbidity and mortality, partly due to the disease’s marked biological heterogeneity. Identification of reliable biomarkers associated with metastatic progression is essential for improving risk stratification and clinical management. Matrix metalloproteinase-2 (MMP-2) and routine hematological and metabolic parameters have been implicated in cancer progression, but their roles in breast cancer metastasis remain inconsistent across populations.

**Methods:**

This cross-sectional study evaluated general, hematological and metabolic parameters in breast cancer patients from Dr. Kariadi General Hospital, the main referral hospital for Central Java, Indonesia. Associations with metastasis were assessed using univariate analyses, followed by least absolute shrinkage and selection operator (LASSO) logistic regression for variable selection. Multicollinearity was assessed using variance inflation factor (VIF), and selected variables were entered simultaneously into a multivariable binary logistic regression model. Associations between selected hematological parameters and estrogen receptor (ER) and progesterone receptor (PR) status were also examined. Associations between clinicopathological variables and histologic grade were analyzed using the Kruskal–Wallis test.

**Results:**

Histologic grade was the only general characteristic significantly associated with metastasis (*P* < 0.001). In univariate analysis, metastatic patients showed significantly higher MMP-2, WBC count, and SGOT levels, whereas lower MCV and uric acid levels were detected. Multivariable logistic regression identified MMP-2 (*P* = 0.015; OR = 1.124, 95% CI: 1.023–1.236) and platelet count (*P* = 0.047; OR = 0.973, 95% CI: 0.947–1.000) as independently associated with breast cancer metastasis. Platelet count was also significantly associated with ER status (*P* = 0.039), but not with PR status (*P* = 0.054). Kruskal–Wallis analysis demonstrated significant differences in MMP-2 and uric acid levels across histologic grades I–III.

**Conclusion:**

Elevated MMP-2 levels and reduced platelets were independently associated with breast cancer metastasis, while platelets were additionally associated with ER status. These findings suggest that MMP-2 and platelet count may serve as potential biomarkers associated with metastatic behavior and hormone receptor status in breast cancer.

## 1 Introduction

Breast cancer remains the most prevalent cancer among women in Indonesia, accounting for 66,271 (16.2%) total cases and 22,598 (9.3%) deaths nationwide (1). The incidence and mortality of female breast cancer in 2025 were projected to increase by 35.19% and 49,79% in 2050, respectively (2). The economic burden due to breast cancer patients reaches US$46 million in 2020, with highest total annual cost per patient incurred by age group of 50–54 (US$1,150) (3). These trends highlight the urgent need for improved prevention services and timely diagnosis of breast cancer in Indonesia, especially in monitoring tumor aggressiveness and metastatic potential.

Breast cancer susceptibility and progression are highly influenced by a complex interplay of environmental, lifestyle, reproductive, metabolic, and genetic factors (4–6). Several genes and proteins have been identified linked to the breast tumor initiation and progression, including BRCA1/2, HER2, EGFR, c-Myc and Ras gene family (*H-ras*, *K-ras* and *N-ras*) (7–9). While genetic variation influences the oncogenic pathways contributing to tumor initiation (10–13), the disease progression and metastasis are strongly influences by physiological dynamics within tumor microenvironment, including enzymatic degradation of extracellular matrix (ECM) and angiogenesis activation (14).

Among ECM-remodeling enzymes, matrix metalloproteinase (MMP) plays a pivotal role in remodeling type IV collagen of the basal membrane (14). Two most crucial circulating MMPs reflecting tumor aggressiveness are MMP-2 and MMP-9. MMP-2—a zinc-dependent endopeptidase—is known to promote tumor invasion and metastatic spread by disrupting basement membrane and activating angiogenic factors, whereas MMP-9 reflects inflammatory responses during tumor invasion (14–16). Thus, elevated circulating MMP-2 levels in breast cancer patients further demonstrate its direct involvement in remodeling cancer-associated tissues (17,18), while a significant reduction in plasma MMP-2 concentrations following surgical removal of the primary tumor suggests its potential utility as an indicator of successful tumor eradication (19). Collectively, circulating MMP-2 level may potentially serve as a diagnostic and prognostic indicator of breast cancer, reflecting tumor-related proteolytic activity in breast cancer progression.

Despite these promising findings, research on MMP-2 in breast cancer progression among Southeast Asian populations, particularly within Indonesian cohorts, remains limited. To date, only one study, published in Indonesian, has reported relevant cases from population in Makassar, South Sulawesi (20), thereby limiting broader international accessibility and cross-population comparison. Other studies, if any, primarily reported MMP-2 association with cancer progression in different types of cancer rather than breast cancer particularly (21–23). Hence, the study in Indonesia cohort could be beneficial to obtain an important insight about the dynamic level of plasma MMP-2 in breast cancer metastasis case over different population.

In addition, MMP-2 activity is tightly related to systemic inflammatory and hematological parameters alteration, reflecting systemic physiological alterations that accompany metastatic progression. Thus, hematological parameters including red blood cell (RBC), white blood cell (WBC), platelet count, and hemoglobin (Hb) indices may reflect systemic alterations associated with proteolytic pathways involved in tumor dissemination (24–27). Biochemical indicators of tissue damage and metabolic stress could also be related to metastatic activity (27). Given the importance study of these parameters in monitoring cancer progression, nevertheless, the relationship between circulating MMP-2 levels and routine hematological parameters in breast cancer metastasis has not been characterized yet in Indonesian patients.

Therefore, this cross-sectional study aimed to investigate the association between circulating MMP-2 levels and its relationship with hematological and metabolic variables to breast cancer metastasis among patients in Central Java, Indonesia. By integrating molecular biomarkers, including MMP-2, with routine clinical laboratory data, this study seeks to identify potential predictors of metastatic disease and generate locally relevant evidence to support earlier detection strategies and improved monitoring of breast cancer progression in the Indonesian population.

## 2 Materials and methods

### 2.1 Data collection

Patient data for this study were obtained from the Ca Mammae Center at Dr. Kariadi Central General Hospital in Central Java, Indonesia. Data analysis was conducted by the Bioinformatics and Data Science Research Center (BDSRC) in Jakarta, Indonesia. The ethical concerns of this study have been reviewed and approved by the Ethics Committee at RSUP Dr. Kariadi Semarang. The study cohort consisted of 80-woman patients of breast cancer, comprising 40 patients for both group with metastasis and non-metastasis. In the non-metastatic group, primary tumor size ranged from T2 to T4. In contrast, all patients in the metastatic group presented with T4-stage tumors. Pathological lymph node involvement in both groups ranged from N0 to N2. Among non-metastatic breast cancer patients, 37.5% were classified as N0, 57.5% as N1, and 5% as N2. In the metastatic group, 2.5% of patients were classified as N0, while the majority were N1 (72.5%) and N2 (25%). A total of 24 variables related to patient demographics and blood test results were evaluated. Pathological anatomy diagnoses were categorized into Grade I (23.75%), Grade II (41.25%), Grade III (23.75%), and Mixed/NOS (11.25%).

Serum levels of matrix metalloproteinase-2 (MMP-2) were measured using a sandwich enzyme-linked immunosorbent assay (ELISA) with a total MMP-2 conjugate kit (polyclonal anti-human MMP-2, horseradish peroxidase-conjugated), and absorbance was read using a microplate reader (ELx800). Hematological parameters, including hemoglobin (Hb), hematocrit (Ht), red blood cell count (RBC), white blood cell count (WBC), platelet count, mean corpuscular volume (MCV), mean corpuscular hemoglobin (MCH), and mean corpuscular hemoglobin concentration (MCHC), were analyzed from EDTA-anticoagulated whole blood using an automated hematology analyzer (Sysmex XN-1000). Biochemical parameters, including serum glutamic oxaloacetic transaminase (SGOT), serum glutamic pyruvic transaminase (SGPT), urea, creatinine, blood glucose, and uric acid, were measured from serum samples using an automated chemistry analyzer (Atellica). Plasma D-dimer levels were measured from citrated plasma using a coagulation analyzer (Sysmex CN-3000).

### 2.2 Statistical analysis

This study employed both univariate and multivariate analysis with statistical tests. Univariate analyses were first conducted to describe general patient characteristics. Categorical variables were analyzed using the corrected chi-squared $x^{2}=\frac{x^{2}\left(N-1\right)}{N}$ to account for the small sample size. Fisher’s Exact Test was used to assess the low cell count and the Freeman-Halton extension of Fisher’s Exact Test was applied to handle contingency tables larger than 2×2 .

The hematological and metabolic parameters were also examined through univariate analysis. Hemoglobin (Hb), hematocrit (Ht), red blood cell (RBC) count, mean corpuscular hemoglobin concentration (MCHC), and platelet count were observed using the independent t-test. While the non-normally distributed variables, including matrix metalloproteinase 2 (MMP-2), systolic and diastolic blood pressure, mean corpuscular volume (MCV), mean corpuscular hemoglobin (MCH), leucocyte count, serum glutamic-oxaloacetic transaminase (SGOT), serum glutamic-pyruvic transaminase (SGPT), urea, creatinine, blood glucose and uric acid, were analyzed using the Mann-Whitney U-test.

Normally distributed parameters were presented as mean values with 95% confidence intervals (CI), whereas non-normally distributed parameters were presented as medians with interquartile ranges (IQR). Multivariate analysis was performed using LASSO logistic regression for variable selection. Variables retained through Lasso were subsequently evaluated for multicollinearity, and only those with a variance inflation factor (VIF) < 25 were passed to the binary logistic regression to identify the predictors of metastasis. Statistical significance in this study was defined as P<0.05 (two-tailed).

## 3 Results

### 3.1 General characteristics of breast cancer patients

The general characteristics of breast cancer patients are presented in Table 1. Variables examined in this study included histologic grade, age, BMI, age at menarche, breastfeeding history, family history of breast cancer, and history of benign breast disease of the patients. The histologic grade was grouped as grade I, grade II, grade III, and mixed or not otherwise specified (NOS). The patient’s age was analyzed as a continuous variable. In the metastasis group, ages ranged from 31 to 46 years, whereas in the non-metastasis group, ages ranged from 32 to 66 years. The body mass index (BMI) was classified as underweight (BMI <18.5; 18.75%), normal (18.5 < BMI <25; 68.75%) and overweight (BMI ≥ 25; 12.5%). Age at menarche was grouped as < 12 years (16.25%) and ≥ 12 years (83.75%). A history of breastfeeding was reported in 56.25% of breast cancer patients. Family history of breast cancer was present in 26.25% of patients, while 28.75% had a history of benign breast tumor. Among these general characteristics of patients, only histologic grade that identified associated ( P<0.001 ) with breast cancer metastasis.

**TABLE 1:** Patient general characteristics.

Variable	Metastasis		P-value
	Not found (n = 40)	Found (n = 40)	
Histologic grade			
G I	1 (2.50%)	18 (45.00%)	<0.001*
G II	25 (65.50%)	8 (20.00%)	​
G III	12 (30.00%)	7 (17.50%)	​
Mixed or NOS	2 (5.00%)	7 (17.50%)	​
Age (years)			
Mean (CI 95%)	48.55 (45.49–51.60)	48.55 (45.63–51.47)	1.000
BMI			
<18.5	5 (12.50%)	10 (25.00%)	0.200
18.5≤x<25	28 (70.00%)	27 (67.50%)	​
≥25	7 (17.50%)	3 (7.50%)	​
Menarche age (years)			
<12	10 (25.00%)	3 (7.50%)	0.065
≥12	30 (75.00%)	37 (92.50%)	​
Breastfeeding history			
No	16 (40.00%)	19 (47.50%)	0.654
Yes	24 (60.00%)	21 (52.50%)	​
Family history			
No	29 (72.50%)	30 (75.00%)	1.000
Yes	11 (27.50%)	10 (25.00%)	​
History of benign breast disease			
No	32 (80.00%)	25 (62.50%)	0.141
Yes	8 (20.00%)	15 (37.50%)	​

Age presented as mean values with 95% CI.

* Considered as statistically significant (P-value <0.05).

### 3.2 Univariate analysis of clinicopathological parameters

This study also investigates clinicopathological variables that include hematological and metabolic parameters, in relation to breast cancer metastasis. The variables analyzed were MMP-2, systole, diastole, Hb, Ht, RBC, WBC, platelet count, MCV, MCH, MCHC, SGOT, SGPT, urea, creatinine, blood sugar, and uric acid. The results of the statistical analyses done with independent T-test or Mann-Whitney U-test of these variables are shown in Table 2. Breast cancer patients with metastasis had significantly higher levels of MMP-2 (441.25 *ng/mL*; P<0.001 ), WBC ( $7.98\times 10^{3}\mu L$ ; P=0.002 ), and SGOT (30 *U/L*; P=0.005 ). In contrast, significantly lower uric acid levels (3.25 *mg/dL*; P=0.021 ) were observed in patient with metastasis.

**TABLE 2:** Clinicopathological variables in breast cancer patients.

Variable	Metastasis		P-value
	Not found (n = 40)	Found (n = 40)	
MMP-2 (*ng/mL*)	238.05 (224.90–263.20)	441.25 (383.08–628.20)	<0.001^b^ ^,^ *
Systole (*mmHg*)	123.40 (121.56–125.24)	125.00 (120.75–128.00)	0.518^b^
Diastole (*mmHg*)	80.00 (76.00–81.00)	80.00 (75.00–81.25)	0.723^b^
Hb ( g/dL )	11.53 (10.98–12.07)	11.21 (10.64–11.77)	0.418^a^
Ht (%)	35.18 (33.57–36.78)	34.62 (32.91–36.33)	0.632^a^
RBC ( μL )	4.20 (3.99–4.41)	4.04 (3.84–4.24)	0.276^a^
WBC ( x1000 μL )	5.99 (5.35–6.63)	7.98 (5.63–14.20)	0.002^b^ ^,^ *
Platelets ( x1000 μL )	309.05 (277.58–340.52)	287.30 (249.34–325.26)	0.375^a^
MCV (f L )	86.75 (83.18–89.40)	86.38 (84.47–88.30)	0.276^b^
MCH ( pg )	28.20 (26.58–30.18)	28.17 (27.34–29.00)	0.791^b^
MCHC ( g/dL )	32.34 (31.89–32.79)	32.56 (32.02–33.10)	0.524^a^
SGOT (*U/L*)	27.00 (22.00–30.25)	30.00 (27.75–32.00)	0.005^b^ ^,^ *
SGPT (*U/L*)	25.65 (22.93–28.37)	28.5 (20.00–31.25)	0.291^b^
Urea (*mg/dL*)	21.00 (19.00–28.00)	26.00 (20.75–29.25)	0.061^b^
Creatinine (*mg/dL*)	0.75 (0.60–0.90)	0.82 (0.76–0.89)	0.336^b^
Blood sugar (*mg/dL*)	104.00 (87.00–130.25)	100.00 (90.50–132.00)	0.862^b^
Uric acid (*mg/dL*)	3.82 (3.67–3.96)	3.25 (2.88–4.20)	0.021^b^ ^,^ *

^a^ Independent T-test, data presented as mean values with 95% CI.

^b^ Mann-Whitney U-test, data presented as median values with IQR.

* Considered as statistically significant (P-value <0.05).

### 3.3 Association of ER and PR status with hematological parameters

For further analysis, hemoglobin (Hb; <12 *g/dL* vs. ≥12 *g/dL*), hematocrit (Ht; <36 % vs. ≥36 %), platelet count ( $\leq 450\times 10^{3}\mu L$ vs. $>450\times 10^{3}\;\mu L$ ) and white blood cell count (WBC; $\leq 10^{4}\mu L$ vs. $>10^{4}\mu L$ ) were categorized to evaluate their associations with estrogen receptor (ER) and progesterone receptor (PR) status. As shown in Table 3, there is no significant association between Hb, Ht, or WBC with the ER and PR status. In contrast, platelet count was significantly associated with ER status ( P=0.039 ), whereas its association with PR did not reach statistical significance ( P=0.054 ).

**TABLE 3:** Association of ER and PR status with hematological parameters (HB, HT, Platelets, WBC).

Variable	Total patients	Hb $\left(g/dL\right)$		P-value	Ht $\left(\%\right)$		P-value	Platelets count $\left(x1.000\;\mu L\right)$		P-value	WBC count $\left(x1.000\;\mu L\right)$		P-value
		<12	≥12		<36	≥36		≤450	>450		≤10	>10	
ER	​	​	​	0.541	​	​	0.801	​	​	0.039*	​	​	0.864
Negative	31	16	15	​	16	15	​	31	0	​	24	7	​
Positive	49	30	19	​	28	21	​	42	7	​	40	9	​
PR	​	​	​	0.234	​	​	0.982	​	​	0.054	​	​	0.697
Negative	41	22	19	​	22	19	​	40	1	​	34	7	​
Positive	39	24	15	​	22	17	​	33	6	​	30	9	​

* Considered as statistically significant (P-value <0.05).

### 3.4 Association of clinicopathological variables with hematologic grade

**FIGURE 1:**
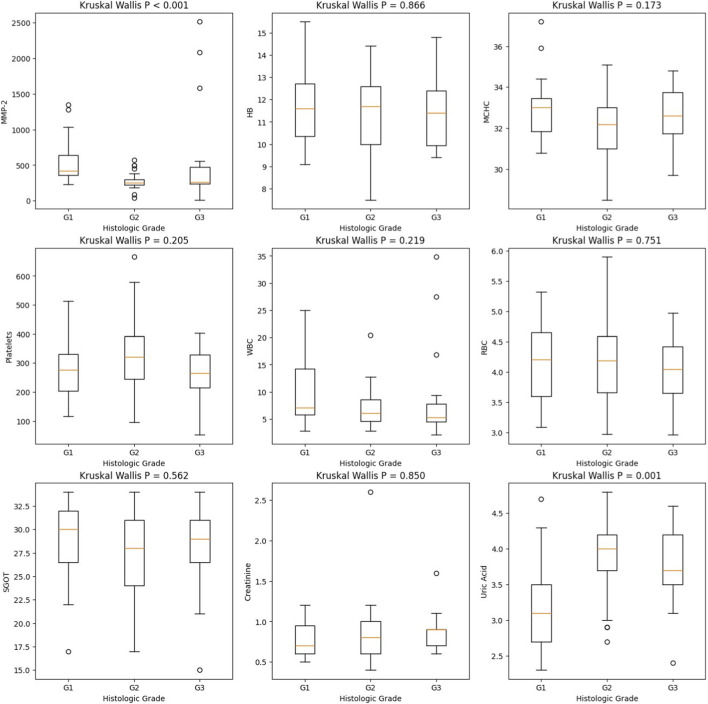
Kruskal Wallis results of association between clinicopathological parameters and hematologic grade.

Furthermore, a Kruskal–Wallis analysis was performed to investigate the association between clinicopathological variables with histologic grade. In this analysis the grades of mixed or NOS were excluded, therefore only grades I, II, and III were analyzed. There are nine variables including MMP-2, Hb, MCHC, platelets, WBC, RBC, SGOT, creatinine, and uric acid levels that were examined in this analysis. The results are visualized using boxplots presented in Figure 1. Among the variables analyzed, only MMP-2 ( P<0.001 ) and uric acid levels ( P=0.001 ) that showed significant differences across histologic grades. The result marks the median MMP-2 in G1 (418.9 *ng/mL*) significantly decreased to G2 (250.5 *ng/mL*) and increased to G3 (258.1 *ng/mL*). In contrast, the median of uric acid levels increased significantly from G1 (3.1 *mg/dL*) to G2 (4 *mg/dL*) before it declined to G3 (3.7 *mg/dL*).

### 3.5 Multivariate analysis

In the multivariate analysis, all general and clinicopathological parameters were initially screened using LASSO logistic regression. As results, MMP-2, age, systole, diastole, MCV, MCH, platelets count and blood sugar are selected. To avoid multicollinearity, the selected variables were further evaluated using VIF <25. Finally, MMP-2, MCV, MCH and platelet count were included in a binary logistic regression model to identify independent risk factors for breast cancer metastasis.

As shown in Table 4, MMP-2 ( P=0.015 ) and platelet count ( P=0.047 ) were independently associated with breast cancer metastatic. MMP-2 demonstrated a positive association with breast cancer metastasis, with OR of 1.124 (95% CI: 1.023–1.236), indicating that each 1 ng/mL increase in MMP-2 results in a 24% increase in the odds of metastasis. On the other hand, platelet count showed negative association with breast cancer metastasis (OR = 0.973; 95% CI: 0.947–1.000), suggesting that each increase of 1000 μL platelet count results in a 2.7% decrease in the odds of metastasis.

**TABLE 4:** Multivariate analysis of clinicopathological parameters to breast cancer metastasis.

Variable	OR	CI 95% (Lower–Upper)	P-value
MMP-2 (ng/mL)	1.124	1.023–1.236	0.015*
MCV (f L )	0.738	0.422–1.290	0.286
MCH ( pg )	0.922	0.251–3.387	0.903
Platelets ( x1000 μL )	0.973	0.947–1.000	0.047*

* Considered as statistically significant (P-value <0.05).

## 4 Discussion

Breast cancer diagnosis and prognosis remain challenging due to the tumour’s marked heterogeneity, characterized by a wide range of morphological and molecular subtypes. Identifying reliable biomarkers is therefore a critical step in the development of effective diagnostic and therapeutic strategies. Numerous studies have demonstrated the role of MMPs in tumour initiation, progression, staging, and grading (18,27,28). In particular, the expression of MMP-2 on tumour cells enables the tumour cell to penetrate the extracellular matrix (ECM) and basal membrane (29), which alters architecture of the cell and tissue microenvironments (30). Therefore, this study further examined the role of MMP-2 levels in breast cancer patients from Central Java, Indonesia—an understudied population in molecular oncology—to assess its potential as a biomarker reflecting tumour aggressiveness.

Notable associations between MMP-2 levels and breast cancer metastasis were observed in both univariate ( P<0.001 ) and multivariate ( P<0.015 ) analysis results. These findings clinically suggest that elevated MMP-2 serum levels are independently associated with the elevation of breast cancer metastasis risk in studied population. These outcomes support the biological role of MMP-2 in promoting tumour invasion through ECM remodelling and facilitating tumour cells escape from the ECM and basement membrane barrier (31–33). At the cellular level, this process is closely linked to invadopodia formation, which are actin-rich membrane protrusions utilized by invasive cancer cells to degrade the ECM by focalize proteolytic activity at the tumour-matrix interface (34,35). MMP-2 is one of the key proteases recruiting to these structures during their maturation, enabling localized degradation of basement membrane components and promoting metastatic dissemination (36). As a gelatinase, MMP-2 cleaves type IV collagen—the principal structural component of basement membranes—and further degrades denatured collagen fragments (“gelatin”) generated during initial proteolysis (37).

However, the result in this study contrasts with a prior study conducted in Egyptian breast cancer patients, which reported no significant association of MMP-2 levels between metastatic and non-metastatic group, although MMP-2 levels were still showed significantly higher in breast cancer patients compared with controls (18). These discrepancies may reflect population-specific genetic, environmental, or clinical differences that contribute to MMP-2 expression and activation. Indeed, genetic studies have demonstrated that only two out of 37 specific polymorphisms within the MMP-2 gene are associated with altered cancer susceptibility (38), reflecting that physiological regulation of MMP-2 activity may influence the disease progression rather than mutation incidence alone. From a clinical perspective, the independent association observed in this study suggests that circulating MMP-2 could probably serve as a complementary marker of cancer invasiveness, particularly in studies cohort.

Additionally, MMP-2 plays a significant role in the regulation of blood components, such as increasing platelets activation which can lead to thrombocytopenia (39). Therefore, this study investigated several hematological variables including Hb, Ht, RBC, WBC, MCV, MCH, MCHC, and platelet count. Among these variables, only WBC ( P=0.002 ) and MCV ( P=0.021 ) were significantly associated with metastasis in univariate analysis. In multivariate analysis, platelet count ( P=0.047 ) remained independently associated with breast cancer metastasis. Notably, both Hb and Ht levels were below standard reference ranges in both metastatic and non-metastatic groups, indicating the presence of mild anemia across the study cohort. Although the median WBC values in both groups remained within the normal range ( $4-11\times 10^{3}/\mu L$ ), the upper quartile in the metastatic group reached $14.20\times 10^{3}/\mu L$ , suggesting a tendency toward leukocytosis in a subset of patients. This elevation is more plausibly reflective of tumor-associated inflammation, stress response, or cytokine-driven hematopoietic stimulation rather than primary bone marrow disorders.

Compared with the non-metastatic breast cancer patients, WBC count in group with metastasis was significantly higher (5.99 $\times 10^{3}\;\mu L$ vs. 7.98 $\times 10^{3}\;\mu L$ ). This finding is consistent with previous study that reported WBC count of patients with positive ER/PR status significantly higher compared to controls (40). The systemic inflammation response is commonly triggered during the increasing of cancer aggressiveness, which further activating the cytokine-mediated proteolytic activity and facilitating the degradation of ECM and malignant cell invasion (41,42). While, increasing WBC might indicates indirect local inflammation that supports the metastasis mechanism (43,44).

Moreover, lower MCV values were observed in metastatic group (86.75 fL vs. 86.36 fL), showing similar pattern with a study done with Iranian breast cancer patients (45). The lower MCV in metastatic patients may indicate cancer-related alterations in erythropoiesis in bone marrow or anemia caused by chronic inflammatory during advanced malignancies (46). Inflammatory cytokines could interfere with iron metabolism and erythrocyte maturation, leading to subtle reductions in erythrocyte size (47). Although the absolute difference in MCV between groups was relatively small, this pattern suggests that hematological alterations may reflect systemic physiological stress accompanying metastatic disease rather than serving as a direct mechanistic driver (48).

Interestingly, the present study demonstrates a contrasting pattern with decreasing platelet count in metastatic patients compared with non-metastatic patients (287.3 μL vs. 309.05 μL ). This finding is contrast to the previous reports describing thrombocytosis as a complementary marker of tumour activity and a feature of aggressive disease (49). These results suggest that platelet dynamics may vary according to disease stage, tumor burden, or population-specific factors, and therefore warrant further investigation. Malignant cells can disrupt normal hematopoiesis. Tumor cells may release procoagulant and inflammatory mediators that alter platelet production, activation, and consumption, potentially leading to either thrombocytosis or thrombocytopenia. In breast cancer, reduced platelet count may be associated with sustained activation of the coagulation cascade, resulting in increased platelet consumption and subsequent thrombocytopenia (50). Platelet depletion during metastasis may reflect enhanced utilization in tumour-associated microthrombi formation, chronic systemic activation, bone marrow suppression, or treatment-related effects (51,52).

In complement with MMP-2 and hematological parameters, the metabolic parameters, including SGOT, SGPT, urea, blood glucose, and uric acid also evaluated for their association with the breast cancer metastasis. SGOT levels were observed significantly higher ( P=0.005 ) in breast cancer patients with metastatic. This finding that may be partially explained by the presence of liver metastasis in 27.5% of patients in the metastatic group. Since SGOT alterations have been closely linked to hepatocellular carcinoma progression (53).

In contrast, uric acid levels in metastatic group were found significantly lower ( P=0.021 ) compared with non-metastatic patients (3.25 *mg/dL* vs. 3.87 *mg/dL*). Although some studies suggest high serum uric acid (SUA) levels are associated with a poor survival rate of breast cancer (54,55), others find that low SUA is associated with higher, not lower, cancer incidence, suggesting a complex relationship where too high or too low levels of SUA might be detrimental (56,57). A plausible explanation for the reduced uric acid in our metastatic cohort is that tumor-induced liver destruction may impair the production of enzymes required for purine catabolism, thereby decreasing uric acid synthesis. Furthermore, while uric acid functions as a major extracellular antioxidant, it also exhibits pro-oxidant and pro-inflammatory effects under pathological conditions (58,59). During metastasis, physiological stress induces the consumption of uric acid to protect cells against reactive oxygen species (ROX). Thus, the reduction may indicate increased antioxidant consumption or altered purine metabolism in rapidly proliferating tumors (60).

According to clinical perspective, the independent association between plasma MMP-2 levels and breast cancer metastasis indicates that MMP-2 could probably serve as a complementary biomarker reflecting cancer aggressiveness and remodeling activation of extracellular matrix. Even though the reported odds ratio indicates apparent increase in metastatic risk per unit elevation of MMP-2, it may still support a clinical value when integrated with routine hematological and metabolic parameters. A multiple biomarker application may improve rapid diagnosis of patients with high risk of metastatic progression, particularly in resource-limited clinical settings where advanced molecular equipment is not routinely available.

Furthermore, there are several limitations that should be considered with the analysis results in this study. This study has relatively small sample sizes which may have reduced statistical power and limited the precision of subgroup analysis. In addition, the use of a single-center cohort from Dr. Kariadi Central General Hospital may introduce selection bias and restrict the generalizability of the findings to broader population. Future studies employing larger, multi-center datasets and prospective designs are needed to validate and extend the findings in this study.

## 5 Conclusion

In this study of breast cancer patients from Central Java, Indonesia, MMP-2 emerged as a key biomarker associated with metastatic disease across univariate, multivariate, and histologic-grade–based analyses. Elevated MMP-2 levels were independently associated with increased odds of metastasis, supporting its role in tumor invasion and progression. In contrast to many prior reports, platelet count demonstrated a negative association with metastasis, suggesting potential population-specific or disease-stage–dependent differences in hematological responses. Additionally, alterations in WBC count, MCV, and uric acid levels were observed in metastatic disease, with uric acid showing significant variation across histologic grades.

Overall, these findings emphasize the heterogeneity of breast cancer biomarkers and the importance of evaluating their clinical relevance within specific populations. Further prospective studies with larger cohorts are warranted to validate these associations and to clarify the biological mechanisms underlying the observed differences. In addition, molecular subtypes of breast cancer (ER, PR, and HER2 status) should be considered, as they may contribute to biological heterogeneity in MMP expression and hematological responses.

## Data Availability

The raw data supporting the conclusions of this article will be made available by the authors, without undue reservation.
